# Suggestions for further clarifying the sources of heterogeneity and potential biases in ART and malignancy risk analysis

**DOI:** 10.1097/JS9.0000000000002695

**Published:** 2025-06-12

**Authors:** Fang Dong, Huawei Tian, Yaohui Han

**Affiliations:** a Shenzhen Hospital (Futian) of Guangzhou University of Chinese Medicine, Shenzhen, China; b Chronic Disease Prevention and Treatment Center of Shenzhen Futian District, Shenzhen, China

*Dear Editor,*

We read with great interest the recent meta-analysis by Zhang *et al* (2025) published in the *International Journal of Surgery*, which extensively explored the association between assisted reproductive technology (ART) and the risk of malignancy among offspring^[1]^. The authors are to be commended for their rigorous subgroup and sensitivity analyses addressing important sources of heterogeneity. Nevertheless, several methodological considerations could further strengthen this analysis, and we respectfully suggest a few areas for refinement. We have followed the TITAN Guidelines to declare that no AI was used in this correspondence, to insurance transparency^[2]^.

Firstly, despite comprehensive subgroup analyses based on tumor type, ART treatment modality, geographic region, and publication year, substantial heterogeneity remained largely unexplained. For example, the pooled estimate of overall malignancy risk (OR 1.04, *I*^2^ = 92%) suggests significant variation among included studies. Although subgroup analyses were insightful, some subgroups, such as the North American cohort, still exhibited very high heterogeneity (*I*^2^ = 95%). We recommend conducting meta-regression analyses using continuous covariates, such as median or mean follow-up time (reflecting the cancer detection window), the number of adjusted covariates in major studies (reflecting residual confounding), and the effective sample size (to assess small-study effects beyond funnel plot asymmetry)^[3]^. These quantitative assessments could better elucidate the contributions of these factors to the observed heterogeneity, providing insights beyond basic stratification.

Secondly, bias assessment in included studies could be more comprehensive. Although the Newcastle-Ottawa Scale (NOS) was appropriately applied, relying on NOS scores alone may not capture specific biases inherent in non-randomized evidence, such as misclassification of exposures or outcomes or time-varying confounding. Therefore, employing the ROBINS-I tool, recommended by the Cochrane Collaboration, may be beneficial. This tool systematically evaluates biases across seven domains, potentially enabling readers to better assess whether selective reporting, temporal bias, or differential surveillance could influence conclusions drawn from studies perceived as high-quality^[4]^.

Finally, a meta-analysis could benefit from a deeper exploration of perinatal modifying factors, such as multiplicity, preterm birth, and birth weight abnormalities, all of which are independently associated with childhood malignancies. ART pregnancies are significantly associated with a high prevalence of multiplicity, preterm labor, and abnormal birth weight. However, these key perinatal factors were neither explicitly listed in Table 2 nor analyzed separately. Including subgroup analyses limited to singletons or combined studies adjusted for birth weight and gestational age could help clarify whether the observed cancer risk increase is directly attributable to ART procedures themselves or their perinatal sequelae.

In summary, we believe that addressing these methodological considerations could significantly enhance the robustness of the conclusions. We commend the authors for their valuable contribution to the literature and hope these suggestions stimulate further clarification and deeper understanding of the implications of ART on offspring malignancy risk.

## Data Availability

None.

## References

[1] Zhang L Tang G Sun X Xiao X Ma F. Assisted reproductive technology and risk of malignancy among offspring: a meta-analysis. Int J Surg (London, England) 2025. doi:10.1097/JS9.0000000000002554. 40405863

[2] Agha RAMG Rashid R Kerwan A. Transparency in the reporting of Artificial INtelligence-the TITAN guideline. Premier J Sci 2025;10:100082.

[3] Thompson SG Higgins JP. How should meta-regression analyses be undertaken and interpreted?. Stat Med 2002;21:1559–73. 12111920 10.1002/sim.1187

[4] Sterne JA Hernán MA Reeves BC. ROBINS-I: a tool for assessing risk of bias in non-randomised studies of interventions. BMJ (Clinical Research Ed) 2016;355:i4919. 10.1136/bmj.i4919 PMC5062054 27733354

